# Elucidating the characteristics of* Mx1* and resistance to influenza A virus subtype H1N1 in the newly developed KWM/Hym mice

**DOI:** 10.1186/s42826-022-00138-z

**Published:** 2022-09-08

**Authors:** Hajin Nam, Boyoung Kim, Avishekh Gautam, Yoo Yeon Kim, Eun Sun Park, Jong Sun Lee, Hyung-Joo Kwon, Je Kyung Seong, Jun Gyo Suh

**Affiliations:** 1 Department of Medical Genetics, College of Medicine, Hallym University, Chuncheon, 24252 Korea; 2 Department of Microbiology, College of Medicine, Hallym University, Chuncheon, 24252 Korea; 3 Center for Medical Science, College of Medicine, Hallym University, Chuncheon, 24252 Korea; 4 Laboratory of Developmental Biology and Genomics, College of Veterinary Medicine, and Korea Mouse Phenotyping Center, Seoul National University, Seoul, 08826 Korea

**Keywords:** Influenza A (H1N1) virus, *Mx1* gene, KWM/Hym mice, Alveolitis

## Abstract

**Background:**

Inbred mice have several advantages, including genetic similarity to humans, a well-established gene manipulation system, and strong tolerance to inbreeding. However, inbred mice derived from a limited genetic pool have a small genetic diversity. Thus, the development of new inbred strains from wild mice is needed to overcome this limitation. Hence, in this study, we used a new strain of inbred mice called KWM/Hym. We sequenced the *Mx1* gene to elucidate the genetic diversities of KWM/Hym mice and observed the biological alterations of the Mx1 protein upon influenza A infection.

**Results:**

The *Mx1* gene in KWM/Hym mice had 2, 4, and 38 nucleotide substitutions compared to those in the *Mx1* gene in A2G, CAST/EiJ, and *Mus spretus* mice, respectively. Moreover, the Mx1 protein in KWM/Hym mice had 2 and 25 amino acid substitutions compared to those in the Mx1 protein in CAST/EiJ and *M. spretus* mice, respectively. To elucidate the function of the Mx1 protein, we inoculated the influenza A virus (A/WSN/1933) in KWM/Hym mice. Nine days after infection, all infected KWM/Hym mice survived without any weight loss. Four days after infection, the lungs of the infected KWM/Hym mice showed mild alveolitis and loss of bronchiolar epithelium; however, the pulmonary viral titers of the infected KWM/Hym mice were significantly lower than that in the infected BALB/c mice (2.17 × plaque-forming units mL^−1^).

**Conclusions:**

Our results demonstrate that the KWM/Hym mice are resistant to influenza A virus infection. Further, these mice can be used as a model organism to understand the mechanism of influenza A virus susceptibility.

## Background

The type A influenza virus causes seasonal infections, resulting in illness and death every year. However, mutations in the influenza A virus led to the emergence of the novel H1N1 virus, which caused respiratory diseases in humans and was responsible for the 2009 swine-flu pandemic [1]. The emergence of new influenza virus strains highlights the need to better understand the pathogenic mechanisms of influenza virus-host interactions since the genetic variation of hosts affects the infection of the influenza A virus subtype H1N1 [2].

*Mx* genes exist in almost all vertebrate genomes and serve as a defense against RNA viruses. Mx proteins are evolutionarily conserved in vertebrates, suggesting that they are critical for antiviral defense across species [3]. The interferon-induced GTP-binding protein, Mx1, is one such antiviral protein that restricts influenza viruses in humans and mice, although the effect depends on the virus strain. The mouse *Mx1* (myxovirus resistance protein 1, Mx dynamin-like GTPase 1, and interferon-inducible protein P78) gene encodes the Mx1 protein, an interferon-inducible nuclear protein, that selectively inhibits influenza A and Thogoto viral multiplication [4].

The house mouse is classified into four subspecies (*M. m. domesticus, M. m. musculus, M. m. castaneus,* and *M. m. bacterianus*) based on their biochemical markers, mitochondrial DNA, and other genetic characters [5–7]. The genetic background of the host is critical for susceptibility to influenza A virus infections in mice [8, 9]. Most laboratory inbred mouse (*M. m. domesticus*) strains, including BALB/c, carry nonfunctional *Mx1* alleles because of deletions or nonsense mutations and consequently exhibit high virus susceptibility [10]. The A2G mice have a wild type *Mx1* allele and resistance against influenza and Togota viruses [10, 11]. Interestingly, several wild-derived strains, including M. spretus, PWK/PhJ, and NZO/HILtJ carry a wild-type *Mx1* allele and are highly resistant to the influenza virus [12]. Interestingly, several wild-derived strains, including SPRET/Ei, PWK/PhJ, and NZO/HILtJ carry a wild-type *Mx1* allele and are highly resistant to the influenza virus. However, wild-derived CAST/EiJ mice carrying two amino acid changes in the Mx1 restriction factor exhibit high susceptibility to influenza A [13, 14]. The wild mice captured in South Korea have mixed genetic components of *M. m. molossinus* and *M. m. musculus* [15, 16]. KWM/Hym mice, a new strain developed from the wild mice captured from the Chuncheon city in Korea, show a 96.4% similarity with PWK/Phj in single nucleotide polymorphism analysis [16]. However, the sequence of the *Mx1* gene, the functional features of Mx1 protein, and whether Mx1 can provide resistance to influenza A virus in the KWM/Hym mice have not been reported yet. Hence, in this study, we investigated the influenza A (H1N1) virus susceptibility of KWM/Hym mice to determine if the strain will be useful for studying influenza A virus infection.

## Results

### The Mx1 protein sequence of KWM/Hym mice differed at two sites from Mx1 of CAST/EiJ

**Fig. 1:**
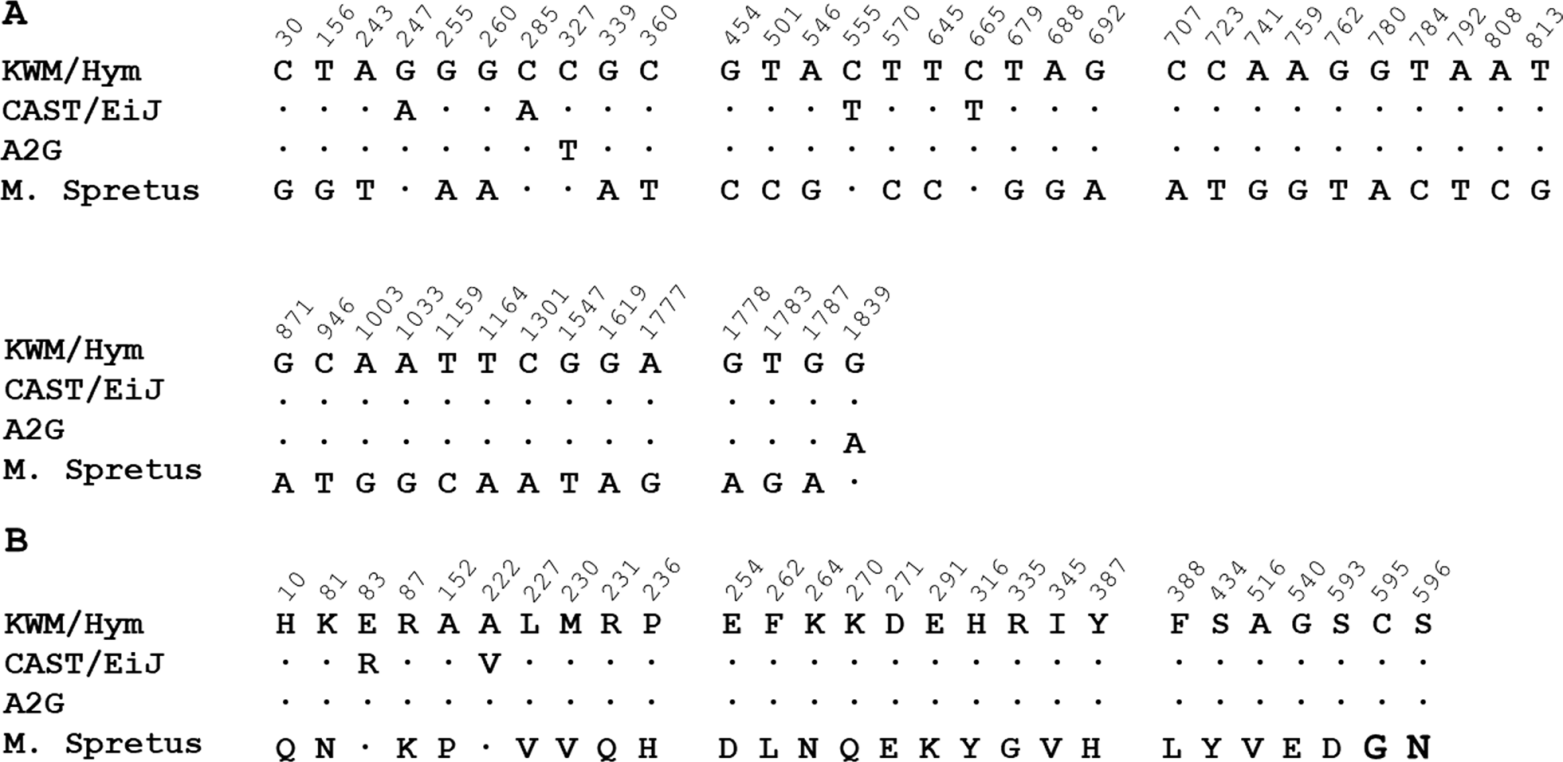
The alignment of nucleotide sequences and amino acids in Mx1 of KWM/Hym mice. **A** The sequence of the *Mx1* gene in KWM/Hym has variation at two positions (327T>C and 1839 A>G) compared to that in A2G mice (*M. m. domesticus*). The sequence of the *Mx1* gene in KWM/Hym has variation with 4 and 38 sites compared to those in CAST/EiJ (*M. m. castaneus*) and *M. spretus* mice, respectively. **B** The KWM/Hym mice have two missense mutations (G83R, A222V) in the G domain of Mx1 protein compared to the protein in CAST/EiJ mice. The Mx1 protein in KWM/Hym compared to the protein in *M. Spretus* has 25 missense mutations, with 13 sites in the G domain, eight in the stalk domain, two in the loop L4 domain, and two in the bundle-signaling element domain. The amino acid sequences were aligned using the T-Coffee Multiple Sequence Alignment Program (https://www.ebi.ac.uk/Tools/msa/tcoffee/)

We sequenced the *Mx1* gene of KWM/Hym mice to determine the functional difference between the *Mx1* gene of the KWM/Hym and CAST/EiJ mice. As shown in Fig. 1, the *Mx1* gene in KWM/Hym mice had four nucleotide substitutions compared to the *Mx1* gene in CAST/EiJ mice (derived from *M. m. castaneus*). The mutations were as follows; G to A at position 247, C to A at position 285, C to T at position 555, C to T at position 665. The sequences of *Mx1* gene had two nucleotide substitutions between the A2G and KWM/Hym mice(T to C at position 327 and A to G at position 1,829). Further, the *Mx1* gene in KWM/Hym mice had 38 nucleotide substitutions compared to the *Mx1* gene of *M. spretus* (Fig. 1A).

The Mx1 protein in KWM/Hym had two amino acid changes compared to the CAST/EiJ mice; G83R (glycine to arginine) and A222V (alanine to valine). The position 83 is located in the G domain of the Mx1 protein and is moderately conserved in vertebrates. The position 222 is also located in the G domain of the Mx1 protein and is highly conserved (Fig. 1B). These two nonsynonymous changes of the Mx1 protein in KWM/Hym mice can cause functional alteration of the Mx1 protein. However, despite two nucleotide substitutions of the *Mx1* gene between A2G and KWM/Hym mice, there are no amino acid substitutions between their Mx1 proteins. Further, there are 25 amino acid substitutions between KWM/Hym and *M. spretus*, indicating that the Mx1 protein of *M. spretus* has an altered function regarding the infection by the pathogenic influenza A virus.

### All KWM/Hym mice survived without weight loss after influenza A virus infection

**Fig. 2:**
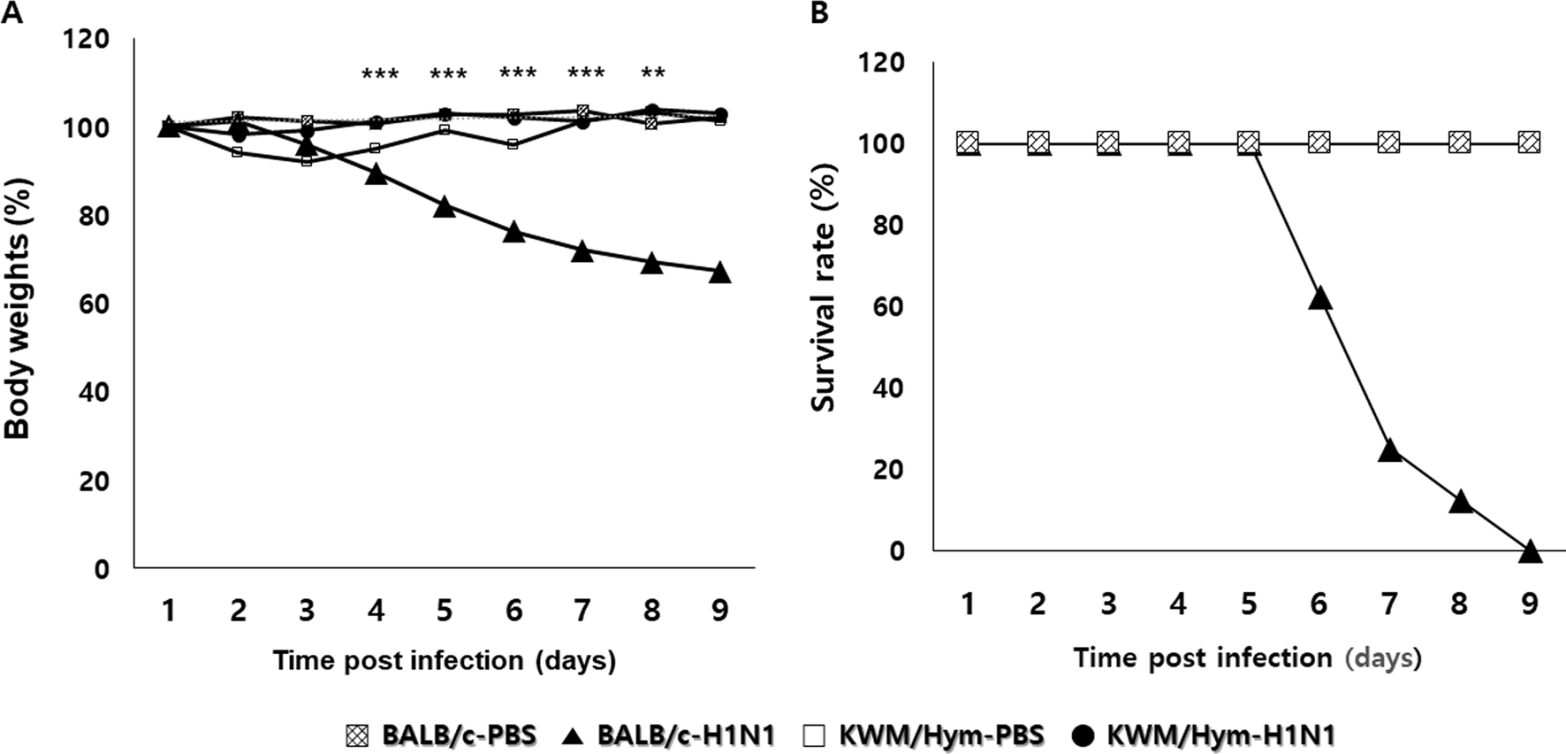
Effect of influenza A virus infection on inbred BALB/c and KWM/Hym mice. The BALB/c and KWM/Hym female mice were challenged intranasally with mouse-adapted A/WSN/1933 virus (H1N1, 1 × 10^5^ pfu/mouse). The body weight (**A**) and the survival rate (**B**) were monitored for nine days (N = 6/group)

To quantify the effect of host genetics on virus susceptibility, BALB/c and KWM/Hym mice were intranasally inoculated with influenza A virus [A/WSN/1933 (mouse-adapted H1N1)]. All infected KWM/Hym mice survived without weight loss until the experiment ceased, nine days after infection (Fig. 2A). In contrast, the infected BALB/c mice showed a significant weight loss compared to the KWM/Hym mice (*p* < 0.001 for days 4–7 and *p* < 0.01 for day 8). The BALB/c mice exhibited 0% survival by day 9 after infection (Fig. 2B).

### Influenza A virus mildly damages lung tissue in KWM/Hym mice

**Fig. 3:**
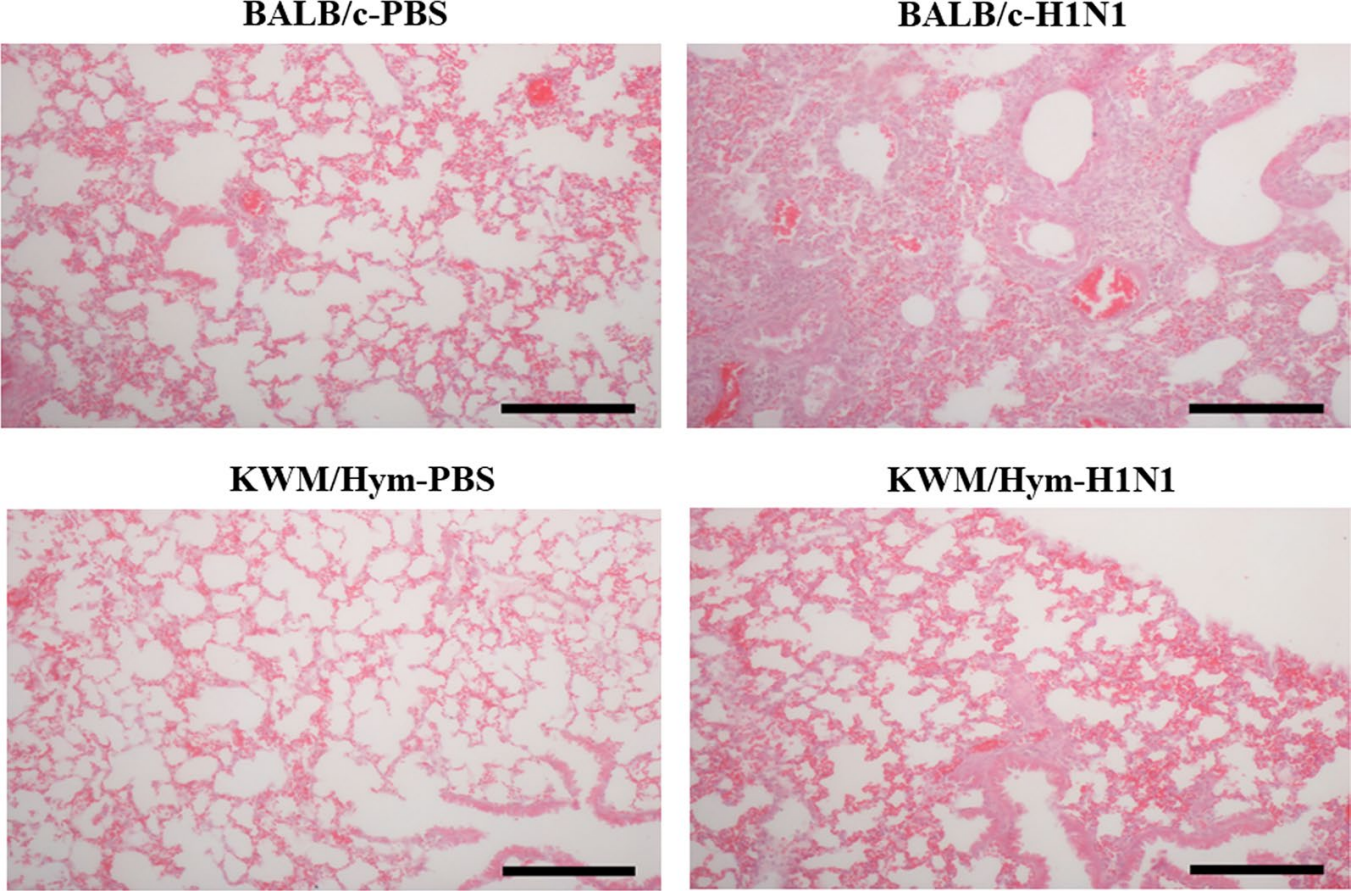
Histopathological lesions in the lung of inbred BALB/c and KWM/Hym mice. The BALB/c and KWM/Hym female mice (N = 6/group) were challenged intranasally with mouse-adapted A/WSN/1933 virus (H1N1, 1 × 10^5^ pfu/mouse). Four days after infection, the lungs were collected, and hematoxylin and eosin staining was performed. The infected KWM/Hym mice showed mild alveolitis and loss of bronchiolar epithelium, significantly less than that of the infected BALB/c mice (scale bars = 100 μm)

Excessive inflammation can cause severe lung lesions during influenza A infection. To evaluate the histopathological changes in the lungs of A/WSN/1933-infected mice, the lungs of each group at day 4 post-infection were examined. The lungs of A/WSN/1933-infected BALB/c mice showed severe alveolitis and fragmentation of alveolar walls along with the presence of lymphocytes, neutrophils, and plasma cells. In contrast, the A/WSN/1933-infected KWM/Hym mice showed mild alveolitis and loss of bronchiolar epithelium. No lesions were observed in the lungs of the PBS-treated mice (Fig. 3).

### Influenza A virus does not replicate in the lungs of KWM/Hym mice

**Fig. 4:**
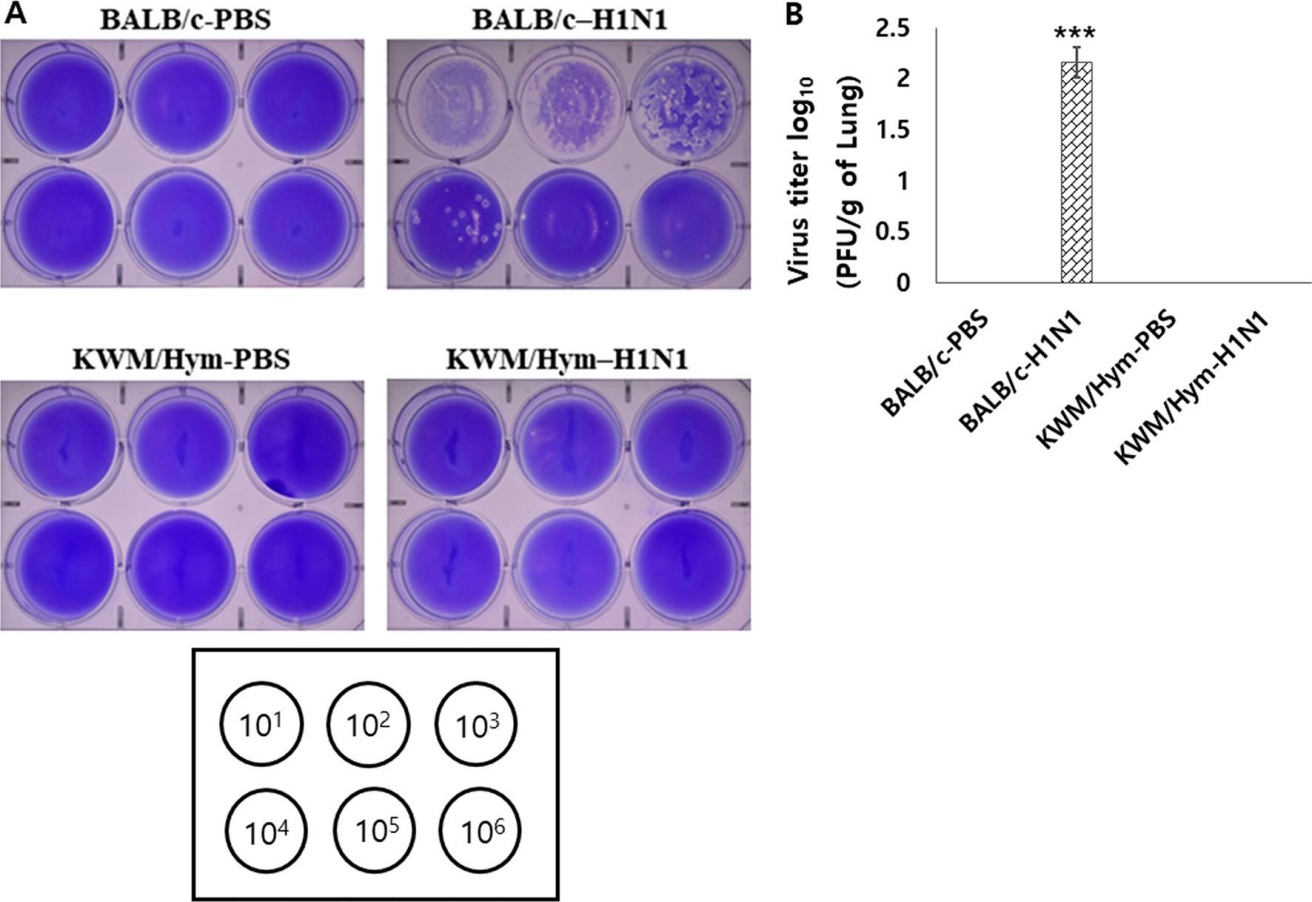
Influenza A virus infection in the lungs of inbred BALB/c and KWM/Hym mice. BALB/c and KWM/Hym female mice were challenged intranasally with mouse-adapted A/WSN/1933 virus (H1N1, 1 × 10^5^ pfu/mouse). Four days after infection, the lungs were collected and homogenized using a Tissue Lyser II. Virus titers were determined using a plaque assay in lung tissue homogenates. The experiment was performed in triplicates using the Madin-Darby Canine Kidney cells (N = 3/group). ***p < 0.0005

To investigate whether the survival rate and histopathological changes in the A/WSN/1933-infected mice were involved in the influenza A virus replication in the lungs, we checked the pulmonary viral titer. To measure virus titers in the lungs of the infected mice, we performed a plaque assay using MDCK cells. The pulmonary viral titers in the infected BALB/c mice (2.17 × pfu mL^−1^) were significantly higher than the titers in the infected KWM/Hym mice (*p* < 0.0005). No plaques were observed in PBS-treated KWM/Hym and PBS-treated BALB/c mice (Fig. 4).

## Discussion

In this study, we sequenced the *Mx1* gene and investigated the susceptibility of the KWM/Hym mice to influenza A virus infection. Sequencing revealed that the KWM/Hym mice carry the wild-type *Mx1* gene. The *Mx1* gene of KWM/Hym mice had 2, 4, and 38 nucleotide substitutions compared to those in the A2G, CAST/EiJ, and *M. spretus* mice, respectively (Fig. 1A). The Mx1 protein in KWM/Hym had two missense mutations compared to the Mx1 protein in CAST/EiJ mice (Fig. 1B). These two missense mutations (G83R, A222V) were located in the G domain of the protein. Further, the Mx1 protein in KWM/Hym had 25 missense mutations compared to the Mx1 protein in *M. spretus*, with 13 sites in the G domain, eight in the stalk domain, two in the loop L4 domain, and two in the bundle-signaling element domain (Fig. 1B). The *Mx1* gene on mouse chromosome 16 has a critical role in influenza A virus infection [17]. The *Mx1* gene is induced by interferon α and β following viral infection. Most inbred mouse strains have alterations in the *Mx1* gene and are susceptible to the influenza A virus [10]. However, some inbred strains (NJL, *Mus musculus*; SPR, *M. spretus*) originating from wild mice have a normal *Mx1* gene and show resistance against the influenza A virus [18, 19]. The loop L4 domain is critical for the binding of Mx1 to influenza A virus [12].

The two positions 83 and 222 on the G domain of Mx1 protein, where we observed amino acid substitutions in the KWM/Hym mice compared to the CAST/EiJ mice, are conserved among vertebrate Mx1 proteins. This suggests that these positions might be important for biological function. To prove our hypothesis, we investigated the resistibility of the KWM/Hym mice to the influenza A virus. As speculated, all influenza A virus-infected KWM/Hym mice survived without weight loss (Fig. 2). The lungs of infected KWM/Hym mice showed mild alveolitis and loss of bronchiolar epithelium on day 4 after influenza A virus infection (Fig. 3). However, the virus was not detected in the lungs of the infected KWM/Hym mice (Fig. 4). These results indicate that KWM/Hym mice have a normal functioning *Mx1* gene and are resistant to infection with the influenza A virus.

An immune response occurs in mice intranasally infected with the influenza A virus [20]. Influenza A virus-reactive IgM and IgG levels in the serum of the influenza A-infected mice decrease and increase, respectively, 5–14 days post-infection [21]. Hence, to confirm the resistance of KWM/Hym mice to the influenza A virus, the involvement of immune responses, such as the serum levels of influenza A virus-reactive IgM and IgG, should be measured 5 to 14 days post-infection. The pathological changes in the lungs of influenza A virus-infected KWM/Hym mice might be restored at a later stage of influenza A virus infection. Since the *Mx1* gene conveys resistance to infection with the Thogoto virus [22], it is interesting to consider whether KWM/Hym mice also have resistance to Thogoto virus.

## Conclusions

Our results indicate that KWM/Hym mice might be useful animals for studying the mechanism of influenza A virus infection. Further, this mouse model may overcome the limitations of the current inbred mouse models that have impaired *Mx1* gene function.

## Methods

### Animals

Eight-week-old female BALB/c (inbred) and KWM/Hym mice were used in this study. BALB/c mice were purchased from DBL (Eumseong, Korea). KWM/Hym mice were generated from Korean wild mice. The mice were maintained in biosafety level (BSL)-2 conditions at 22 ± 2 °C, 55 ± 10% relative humidity, and a 12 h light and 12 h dark cycle. Normal rodent pellet diet (Cargill Agri Purina, Seongnam, Korea) and water were provided ad libitum. For infection, 1 × 10^5^ plaque-forming units (pfu) virus/mouse were inoculated intranasally. Body weight was measured once a day during the experimental period. All animal experimentation, including animal care, was conducted in accordance with the regulations of the Institutional Animal Care and Use Committees of Hallym University (Hallym-2018–56).

### Variant analysis of the *Mx1* gene

Genomic DNA was extracted from the liver of KWM/Hym mice by InstaGene Matrix (Bio-Rad, USA). The primer sequences were as follows: 1F, 5′-GAGTTCTTAAGAACGTCAGAAGG-3′ 1R, 5′-GATACACCAGGTTCCGCATC-3′, 2F, 5′-CAGGAGGTGGACCCTGAAG-3′, 2R, 5′-CGGATCAGGTTTTCAGCTTCC-3′, 3F, v-TGGTCCAAATGCCCTTCGTA-3′, 3R, 5′-AAAGCCACATAGCTAGCCTGG-3′. PCR was carried out with Dr. MAX DNA Polymerase (Doctor protein INC, Korea) and subjected to the following conditions: initial denaturation at 95 °C for 5 min, 35 cycles (95 °C for 30 s, 55–60 °C for 30 s, 72 °C for 60 s), final elongation at 72 °C for 7 min. The PCR products were purified using the Multiscreen filter plate (EMD Millipore Corporation, Billerica, USA). The purified PCR products were sequenced by ABI PRISM 3730XL Analyzer with BigDye (R) Terminator v3.1 cycle Sequencing Kit (Applied Biosystems, Foster City, USA). Variant Reporter Software Version 1.1 (Applied Biosystems) was used to detect variants with three reference sequences (CAST/EiJ: KX774216, A2G: AH0020046, *M. spretus*: KT591117.1). All variants of the *Mx1* gene were confirmed from three individual mice. The nucleotide and amino acid sequences were aligned using the T-Coffee Multiple Sequence Alignment Program (https://www.ebi.ac.uk/Tools/msa/tcoffee/).

### Influenza A virus culture

Influenza A virus [A/WSN/1933 (mouse-adapted H1N1)] was obtained by inoculation in specific-pathogen-free embryonated eggs or infection of the Madin-Darby Canine Kidney (MDCK) cell line [23]. MDCK cells were purchased from American Type Culture Collection (ATCC, Manassas, USA) and maintained in minimum essential media with 10% fetal bovine serum, 100 µg/mL streptomycin, and 100 U/mL penicillin. Virus preparation and experiments were performed under BSL-2 conditions.

### Plaque assay for titration of virus

A plaque assay was performed using the procedures of Gautam et al*.* [21] with modifications. Lungs, harvested four days after intranasal infection, were homogenized using a Tissue Lyser II (Qiagen, Hilden, Germany). The lysates were centrifuged at 13,000 rpm for 5 min at 4 °C and the supernatants were collected. The tenfold serially diluted supernatants were plated onto MDCK-monolayer containing six-well plates, which were previously washed with PBS. The plates were then allowed to stand at room temperature for 1 h, with shaking at 15–20 min intervals. The supernatant was then discarded, and the plates were overlaid with Dulbecco's Modified Eagle Medium/F12 agar (2 mM glutamine, 4% bovine serum albumin, 10 mM HEPES, 2.5% sodium bicarbonate, 50 mg/mL diethylaminoethyl dextran, 1 µg/mL L-tosylamide-2-phenylethyl chloromethyl ketone-treated trypsin, 100 U/mL penicillin, 100 μg/mL streptomycin, and 0.6% immunodiffusion-grade agar). After the agar layer solidified, the plates were incubated at 37 °C for 72 h in a 5% CO_2_ atmosphere. Following incubation, plates were stained with 0.1% crystal violet and inspected for plaques 1 h later.

### Histopathological analysis of lungs

Collected lung sections were fixed in 10% neutral buffered formalin, routinely processed, and embedded in paraffin. Sections were cut to 5 μm sections and stained with hematoxylin and eosin. Histopathological alterations of the lung were examined under an inverted microscope and the images were analyzed using an Axiovision Rel. 4.7 software (Carl Zeiss, Oberkochen, Germany).

### Statistical analysis

Results are presented as the mean ± standard deviation. The statistical significance of differences between the two samples was evaluated using a Student’s *t*-test; P-values less than 0.05 were considered statistically significant.

## Data Availability

The datasets used and analyzed in this study are available from the corresponding author upon reasonable request.

## Abbreviations

ATCC: American Type Culture Collection
BSL: Biosafety level
MDCK: Madin-Darby Canine Kidney
Mx1: Myxovirus resistance protein 1
pfu: Plaque forming units

## References

[1] Dawood FS, Jain S, Finelli L, Shaw MW, Lindstrom S, Garten RJ (2009). Emergence of a novel swine-origin influenza A (H1N1) virus in humans. N Engl J Med.

[2] Ramakrishnan MA, Gramer MR, Goyal SM, Sreevatsan S (2009). A Serine12Stop mutation in PB1-F2 of the 2009 pandemic (H1N1) influenza A: a possible reason for its enhanced transmission and pathogenicity to humans. J Vet Sci.

[3] Verhelst J, Hulpiau P, Saelens X (2013). Mx proteins: antiviral gatekeepers that restrain the uninvited. Microbiol Mol Biol Rev.

[4] Boon AC, deBeauchamp J, Hollmann A, Luke J, Kotb M, Rowe S (2009). Host genetic variation affects resistance to infection with a highly pathogenic H5N1 influenza A virus in mice. J Virol.

[5] Bonhomme F, Catalan J, Britton-Davidian J, Chapman VM, Moriwaki K, Nevo E (1984). Biochemical diversity and evolution in the genus Mus. Biochem Genet.

[6] Yonekawa H, Moriwaki K, Gotoh O, Hayashi JI, Watanabe J, Miyashita N (1981). Evolutionary relationships among five subspecies of Mus musculus based on restriction enzyme cleavage patterns of mitochondrial DNA. Genetics.

[7] Moriwaki K, Shiroishi T, Yonekawa H (1994). Wild mouse from a geneticist’s viewpoint, Genetics in Wild Mice.

[8] Srivastava B, Blazejewska P, Hessmann M, Bruder D, Geffers R, Mauel S (2009). Host genetic background strongly influences the response to influenza a virus infections. PLoS ONE.

[9] Maurizio PL, Ferris MT, Keele GR, Miller DR, Shaw GD, Whitmore AC (2018). Bayesian diallel analysis reveals Mx1-dependent and Mx1-independent effects on response to influenza A virus in mice. G3 (Bethesda).

[10] Staeheli P, Grob R, Meier E, Sutcliffe JG, Haller O (1988). Influenza virus-susceptible mice carry Mx genes with a large deletion or a nonsense mutation. Mol Cell Biol.

[11] Hug H, Costas M, Staeheli P, Aebi M, Weissmann C (1988). Organization of the murine Mx gene and characterization of its interferin- and virus-inducible promoter. Mol Cell Biol.

[12] Verhelst J, Spitaels J, Nurnberger C, De Vlieger D, Ysenbaert T, Staeheli P (2015). Functional comparison of Mx1 from two different mouse species reveals the involvement of loop L4 in the antiviral activity against influenza A viruses. J Virol.

[13] Nurnberger C, Zimmermann V, Gerhardt M, Staeheli P (2016). Influenza virus susceptibility of wild-derived CAST/EiJ mice results from two amino acid changes in the MX1 restriction factor. J Virol.

[14] Shin DL, Hatesuer B, Bergmann S, Nedelko T, Schughart K (2015). Protection from severe influenza virus infections in mice carrying the Mx1 influenza virus resistance gene strongly depends on genetic background. J Virol.

[15] Lee YH, Lee JE, Oh SH, Yun YM, Lee JE, Jin HK (2000). Genetic characteristics of Korean wild mice (*Mus musculus* spp.) by biochemical marker gene. Lab Anim Res.

[16] Nam H, Kim YY, Kim B, Yoon WK, Kim HC, Suh JG (2018). Genetic and morphometric characteristics of Korean wild mice (KWM/Hym) captured at Chuncheon. South Korea Lab Anim Res.

[17] Staeheli P, Haller O (1987). Interferon-induced Mx protein: a mediator of cellular resistance to influenza virus. Interferon.

[18] Jin HK, Yamashita T, Ochiai K, Haller O, Watanabe T (1998). Characterization and expression of the Mx1 gene in wild mouse species. Biochem Genet.

[19] Vanlaere I, Vanderrijst A, Guenet JL, De Filette M, Libert C (2008). Mx1 causes resistance against influenza A viruses in the Mus spretus-derived inbred mouse strain SPRET/Ei. Cytokine.

[20] Dougan SK, Ashour J, Karssemeijer RA, Popp MW, Avalos AM, Barisa M (2013). Antigen-specific B-cell receptor sensitizes B cells to infection by influenza virus. Nature.

[21] Gautam A, Park BK, Kim TH, Akauliya M, Kim D, Maharjan S (2019). Peritoneal cells mediate immune responses and cross-protection against influenza A virus. Front Immunol.

[22] Haller O, Frese M, Rost D, Nuttall PA, Kochs G (1995). Tick-borne thogoto virus infection in mice is inhibited by the orthomyxovirus resistance gene product Mx1. J Virol.

[23] Rhee JW, Kim D, Park BK, Kwon S, Cho S, Lee I (2012). Immunization with a hemagglutinin-derived synthetic peptide formulated with a CpG-DNA-liposome complex induced protection against lethal influenza virus infection in mice. PLoS ONE.

